# Root-knot nematodes demonstrate temporal variation in host penetration

**DOI:** 10.21307/jofnem-2020-037

**Published:** 2020-04-13

**Authors:** Shova Mishra, Peter DiGennaro

**Affiliations:** Department of Entomology and Nematology, University of Florida, Gainesville, FL, 32611

**Keywords:** Root-knot nematodes, Temporal variation, Circadian rhythms, Bioassays, Nematode biology

## Abstract

Root-knot nematodes (RKN; *Meloidogyne* spp.) are obligate plant parasites that require constant communication with their host to establish and maintain specialized feeding cells. The intimacy of this interaction likely requires constant monitoring of host biology and behavior. As plant processes follow tightly regulated circadian and diurnal patterns, RKN may use similar cues to regulate aspects of this symbiosis. We interrogated RKN biology within the context of host diurnal rhythms throughout nematode development. At 24-hr post-inoculation, RKN penetrated host roots significantly more when inoculated during the night compared to the day. We excluded the possibility that this phenomenon is due to nematode perception of light penetrating the soil, as an identical phenomenon is observed under inverted light conditions. Additionally, when plants were allowed to equilibrate and adjust their light-driven clock under constant light conditions, the temporal variation in nematode penetration was abolished. This phenomenon is not present during earlier nematode developmental stages as egg hatch and infective juvenile mobility did not follow rhythmic patterns and are not affected by light. Taken together, it appears nematode host seeking and penetration are at least partially influenced by daily changes in plant root signaling and light does not have a direct effect on RKN developmental stages. Understanding the role and origin of circadian and diurnal rhythms in the plant–nematode interaction underscores the importance of exploiting basal plant biology to develop novel control methods for these pathogens.

Light is a fundamental regulator of plant biology that directs many metabolic, chemical, and physiological processes often manifesting as circadian and diurnal rhythms (Yazdanbakhsh et al., 2011). Circadian rhythms are an endogenous biological cadence with a periodicity of approximately 24 hr and are regulated by an internal clock that synchronizes biological processes with external environmental cues. Diurnal rhythms are distinct in that they are directly influenced by the presence or absence of light. Over 30% of plant *Arabidopsis thaliana* transcriptome is under circadian control (Covington et al., 2008) and 50% of the plant’s transcribed genome is under diurnal control (Michael et al., 2008). As plant processes follow tightly regulated circadian and diurnal patterns, many obligate plant parasites use similar cues to regulate aspects of the plant–pathogen interaction (Oberpichler et al., 2008; Bhardwaj et al., 2011; Wang et al., 2011). Colony formation of the bacterial pathogen *Pseudomonas syringae* pv. tomato DC3000 (Bhardwaj et al., 2011), spore formation, and spore dispersal of fungal pathogen *Hyaloperenosposra arabidopsidis* is regulated by host circadian rhythm (Wang et al., 2011). Attachment and motility of bacterial pathogen *Agrobacterium tumefaciens* to tomato roots follow a diurnal pattern (Oberpichler et al., 2008).

Root-knot nematodes (RKN) infect almost all cultivated plants and are one of the most damaging plant-parasitic nematodes causing devastating agricultural losses (Trudgill and Blok, 2001). These obligate plant parasites form an intimate interaction with their hosts, and directly sample and respond to host plant signals (Curtis, 2008). Hatching of RKN eggs can occur in the absence of host cues; however, root exudates are known to stimulate hatching (Karssen et al., 2013). In the case of *M. chitwoodii* egg hatching is also dependent on the age of the plant; in a young plant, hatching does not require host diffusates while eggs in older plant require root exudate to hatch (Wesemael et al., 2006). Plant volatiles are also often used as signals for infective second-stage juveniles (J2) to locate potential hosts. Once J2 detect compatible environmental and host cues, migration toward the host begins (Kihika et al., 2017). Carbon dioxide is the most common and potent nematode attractant released by living and decaying plant and animal tissues (Perry, 2005). Soil pH and chemical components of root diffusates also determine nematode migration toward host plants (Curtis, 2008). Upon penetration of host roots and migration toward the vascular cylinder, RKN induce the formation of ‘Giant Cells’ (GC) that serve as the sole source of nutrients (Gheysen and Mitchum, 2011). Despite the intimacy of the RKN–plant interaction, these parasites exhibit wide and diverse host ranges, even within species. This parasitic plasticity suggests RKN exploit common or essential mechanisms within host plant biology during successful parasitism.

Circadian and diurnal rhythms also control plant defense responses to pathogen attack (Bhardwaj et al., 2011; Wang et al., 2011; Zhang et al., 2013). Generally, plants are more resistant to pathogens during the day compared to the night suggesting a role for light and dark cycles in informing circadian rhythms (Griebel and Zeier, 2008; Bhardwaj et al., 2011). Much of the Arabidopsis transcriptome is regulated by the circadian and diurnal rhythms and defense response transcripts are among them (Sauerbrunn and Schlaich, 2004; Wang et al., 2011). Plants are programmed to deal with pathogen invasion according to a circadian schedule, even in the absence of pathogen (Zhang et al., 2019). Genes involved in *R-gene* mediated resistance and basal defense are regulated in a circadian manner (Zhang et al., 2013). This rhythmic expression of defense genes could be for functional coordination to prime the regulation at certain time of the day when an infection is most likely. In addition, circadian and diurnal rhythms also act as a determinant of virulence in plant pathogens (Donofrio and Delaney, 2001; Oberpichler et al., 2008; Cohen et al., 2017).

Here we elucidate a role of the endogenous plant clock in RKN parasitism. As an obligate parasite, RKN likely monitors host biology, including the changes in plant signaling that accompany circadian and diurnal rhythms to inform its behavior. Determining the role and origin of circadian and diurnal rhythms in the plant–nematode interaction may emphasize the importance of basal plant biology exploitation.

## Materials and methods

### Nematode inoculum and plant material

Root-knot nematode (*M. incognita* and *M. hapla*) were maintained on ‘Rutgers’ and ‘Tropic VFN’ tomato (*Solanum lycopersicum*), respectively, and cultured under greenhouse conditions with sterile sand. Inoculated plants were maintained under 16 hr light and 8 hr of dark for all experiments. Eggs were extracted from infested roots using 5% household bleach and centrifugal flotation methods (Hussey and Barker, 1973). *Medicago truncatula* cv. Jemalong was used as a model host in all experiments. Seeds of *Medicago* plants were obtained from the US National Plant Germplasm Center. Seeds were sterilized with 70% ethanol and 50% household bleach before planting.

### Nematode penetration bioassays

#### Temporal variation

**Figure 1:**
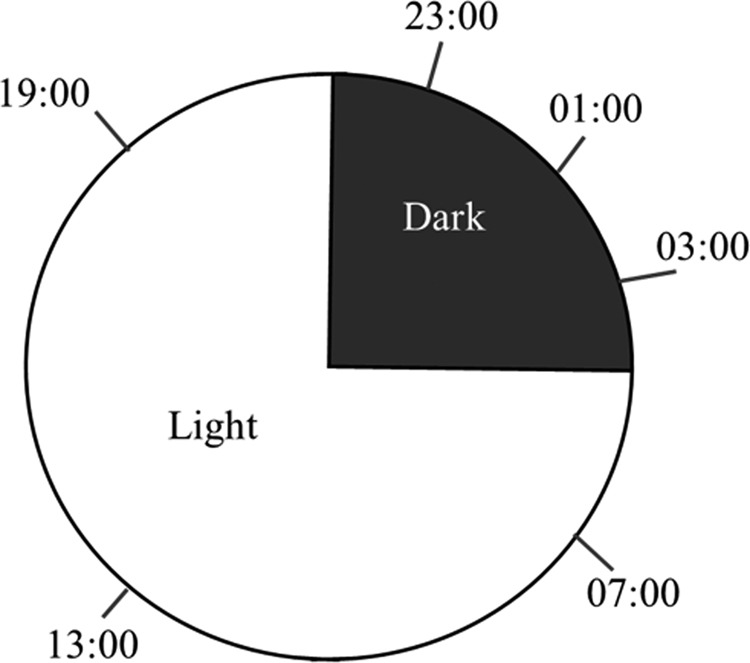
Host plants, *Medicago truncatula* were inoculated with RKN at six different time points; three times points during the light period and three time points in the dark period. In total, 24 hr post-inoculation, root samples were collected at respective time points to check the temporal variation in penetration. All infective juveniles were exposed to host roots for a 24 hr period before quantification.

To examine if temporal variation exists in nematode penetration, we conducted a time-course experiment. Four-week old *M. truncatula* were inoculated with 500 *M. incognita* J2, and plants were maintained under 16 hr of light and 8 hr of dark with a constant maintained temperature of 24°C. Plants were inoculated at six time points: three time points within the 16 hr light period and three time points within the 8 hr dark period (Fig. 1). The experiment was arranged in a completely randomized design with four biological replicates per timepoint. In total, 24 hr after a plant was inoculated, we collected root samples and enumerated nematodes by acid fuchsin staining (Bybd et al., 1983), such that each plant get 24 hr to be in contact with inoculated nematodes. The entire experiment was repeated three times with *M. incognita*. To see if temporal variation exists in another RKN species we also conducted a similar assay using *M. hapla* and repeated the experiment three times.

#### Adjusted light/dark phases

**Figure 2:**
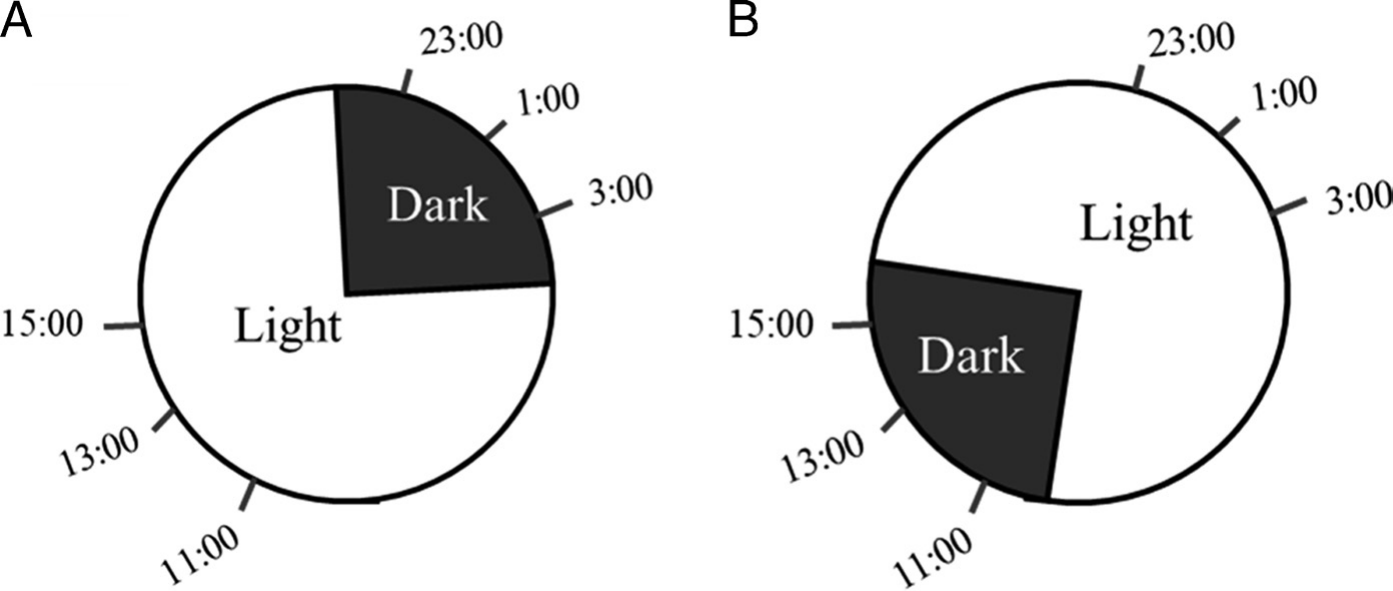
Sampling timepoints for normal (A) and inverted (B) light/dark cycles. To exclude the direct effect of light as a contributing factor to diurnal changes in penetration, we inverted light/dark cycles for equal period of light and dark as in normal condition. Inoculation was done at 2 hr interval for three time points at dark and three time points at light under both conditions. Root samples were collected at 24 hr post-inoculation at respective time points.

Any variation in nematode penetration over a 24 hr period could be attributed to direct light effects on nematode behavior. In order to exclude the effect of this variable and to understand if the temporal difference in penetration is due to the perception of light by nematodes, we altered the length and period plants were exposed to light. Briefly, all plants were inoculated under normal 16 hr light/8 hr dark, these plants were split into two groups. One group stayed in an identical 16 hr light/8 hr dark condition (Fig. 2A), while the other set was immediately transferred to an inverted 16 hr light/8 hr dark phase (Fig. 2B). Plants transferred to the inverted light schedule are not given enough time to equilibrate to the new light signals and do not adjust light-driven biology accordingly. All plants were inoculated with 500 *M. incognita* J2 at six time points with five replications at each timepoint. In total, 24 hr post-inoculation, nematodes were quantified by acid fusion staining as above. The entire experiment was repeated three times.

#### Constant light condition

To further understand if the temporal difference in RKN root penetration is due to the influence of light, we measured nematode behavior under constant light conditions with a constant maintained temperature of 24°C. *Medicago* plants were grown in 16 hr light/8 hr dark. Three-week-old plants were then transferred and trained under constant light for one week, which allows for the plant to equilibrate its circadian and diurnal patterning and inoculated with 500 *M. incognita* J2 at six different time points. After 24 hr post-inoculation, nematode penetration was counted by root staining.

#### Temperature measurement

To exclude diurnal changes in soil temperature as a factor influencing nematode penetration, we recorded soil temperature from four randomly selected plants at each timepoint. Plants were maintained under 16 hr light and 8 hr dark condition with a constant temperature of 24°C. Soil temperature was recorded using temperature probe (Traceable®, Fisherbrand).

### Nematode egg hatching

To examine if the egg hatch of RKN is influenced by the presence or absence of light, we monitored nematode egg hatching in constant light and constant dark conditions for 68 hr. Freshly harvested *M. incognita* eggs were placed on a petri plate and were kept for hatching under constant light and constant dark conditions in incubators for 68 hr. The experiment was maintained at a constant 27°C with four biological replications for each treatment, derived from independently cultured nematode populations. Each replication received 500 nematode eggs. Hatched juveniles were counted at 4-hr intervals for a total of 68 hr. Percent of hatched juveniles at each time points were calculated and used for statistical analysis.

### Nematode mobility assays

To assess the direct effect of light on juvenile mobility, an in vitro experiment was set up.

Second-stage juveniles of *M. incognita* were hatched from freshly collected eggs and 300 J2 were placed on a screen of 60.3 μm pore size set up as a modified Baermann method under constant light and constant dark conditions in incubators. This mesh size allows J2 to migrate through while restricting passive egg movement. The experiment was set up at a constant 27°C with three replications for each treatment. After 4 hr interval, juveniles that migrated through the screen were counted.

### Data analyses

To calculate a percentage of egg hatch, the number of juveniles counted was divided by total number of the eggs kept for hatching. Pairwise comparisons using a *t*-test (*α* = 0.05) were conducted to detect statistical differences in the percentage of egg hatch between consecutive time points and between constant light and constant dark conditions. Also, to detect differences in juvenile mobility between constant light and constant dark condition, a paired *t*-test was used.

Penetration bioassays and temperature data were subjected to one-way analysis of variance (ANOVA) in Statistical Analysis System (SAS Institute Inc. Cary, NC) using Proc Glimmix following negative binomial distribution and normal distribution, respectively. Means were separated using the Tukey test.

## Results

### Penetration bioassays reveal host-driven diurnal changes

**Figure 3:**
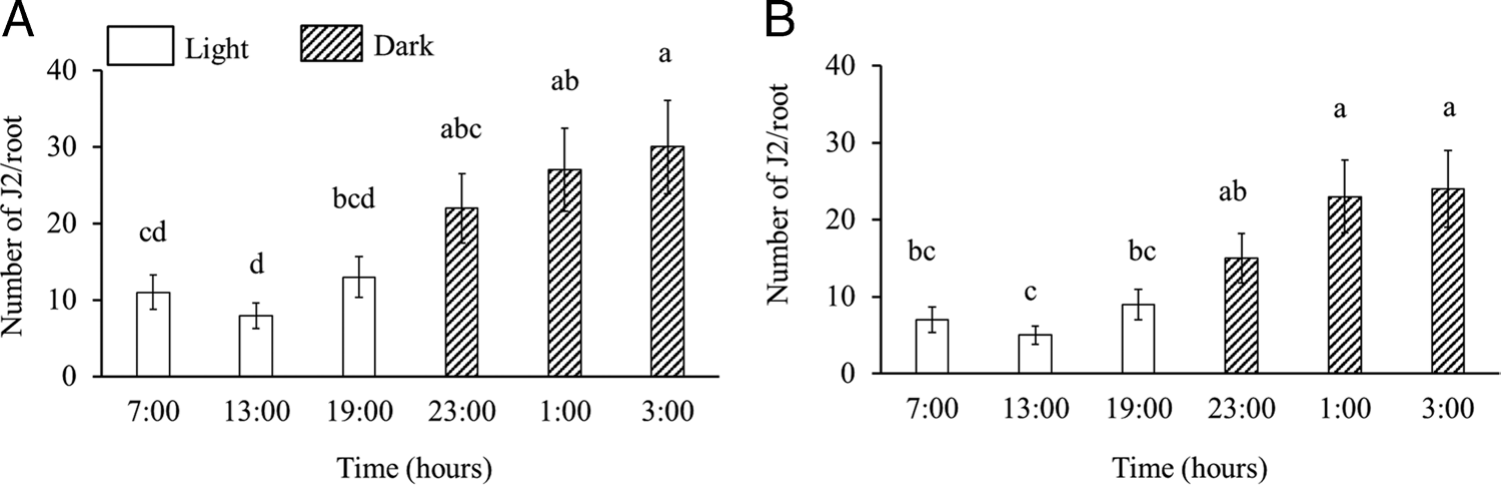
RKN displays temporal variation in penetration with the highest penetration at night, 3:00 a.m. *M. truncatula* was inoculated with *M incognita* (A) and *M. hapla* (B) at six time points, three times points light and three timepoints dark. In total, 24-hr post-inoculation root samples were collected at respective time points. Bars represent standard error and columns with different letters are significantly different (*p* < 0.05). Data shown in each figure represent three independent experiments with four replications per experiment.

**Figure 4:**
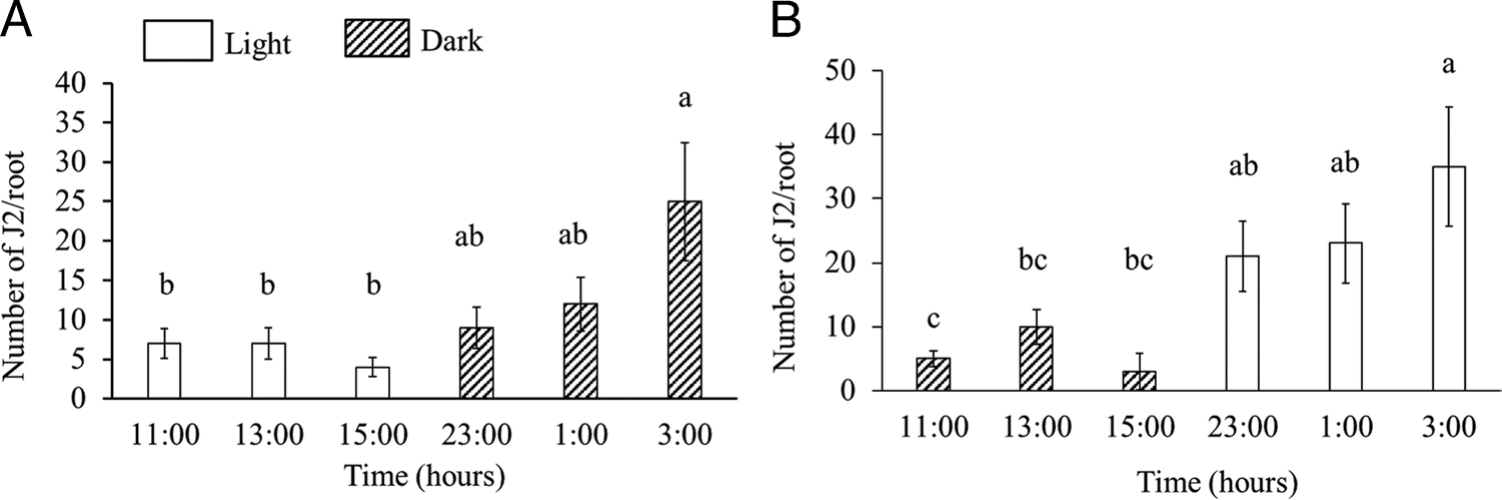
*M. truncatula* plants maintained under normal 16 hr day/8 hr night conditions were inoculated with *M. incognita* at six time points. One set of plants was kept in normal light condition (A) and another set of plants were immediately transferred to an inverted light condition (B). In total, 24 hr post-inoculation root samples were collected at respective time points and nematode inside root was quantified. Bars represent standard error and columns with different letters are significantly different (*p* < 0.05). Data shown in each figure are from three experiments with five replications per experiment.

**Figure 5:**
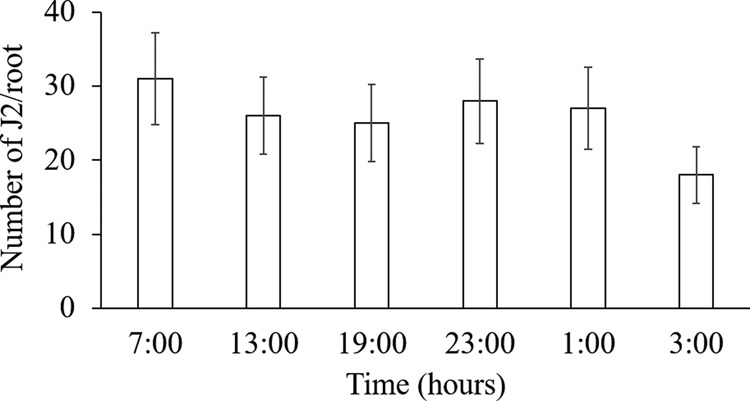
RKN do not exhibit temporal difference in penetration when plants are entrained under constant light conditions. Three weeks old *M. truncatula* plants were kept in constant light for one week. Plants were inoculated with *M. incognita* at six time points. In total, 24 hr post-inoculation root samples were collected at respective time points and nematode inside root was counted. Bars represent standard error. No timepoint was significantly different (*p* > 0.05). Data shown in each figure are from two experiments with four replications in each experiment.

To examine if RKN penetration is influenced by host biology, we inoculated *Medicago* plants at six different time points, three time points during the day (light) and three during the night (dark). All nematodes had an equal 24 hr contact with host plants and we observed that *M. incognita* (Fig. 3A) penetration was higher at dark compared to light (*p* < 0.05). We observed the similar phenomenon of temporal difference when we inoculated *Medicago* plants with *M. hapla* (Fig. 3B). A consistent variation in penetration with time of inoculation was observed in six independent experiments. This temporal variation could be attributed to the direct effect of light or temperature because these two are chief entraining environmental signals (Zeitgebers) that can reset the endogenous timing system (Millar, 2004; Salome and McClung, 2005). To exclude the direct effect of light potentially penetrating the soil and influencing RKN behavior, we inoculated host *Medicago* at different timepoints of the day under normal and an inverted light/dark cycle and sampled after 24 hr allowing the plants to be under inverted light/dark cycles for only 48 hr (Fig. 2). Inverting the light/dark cycle for 48 hr does not allow the plant host to recalibrate its diurnal rhythms (Bhardwaj et al., 2011). A consistent variation in host susceptibility was observed under normal (Fig. 4A) and inverted light conditions (Fig. 4B). *Medicago* showed the greatest susceptibility to RKN at dark, regardless of the light/dark cycle. These data point toward the role of host-driven penetration pattern. To further confirm if nematode penetration is influenced by host endogenous rhythm, we inoculated host plants with *M. incognita* entrained under constant light. We did not observe any temporal differences (*p* > 0.05) on RKN penetration under this constant light condition (Fig. 5). In order to omit the potential effect of varying soil temperature on the observed temporal variation in RKN penetration, we measured soil temperature at over the course of the experimental designs. Soil temperature remains uniform through the day and night cycles (data not shown).

### Egg hatch does not show temporal variation

**Figure 6:**
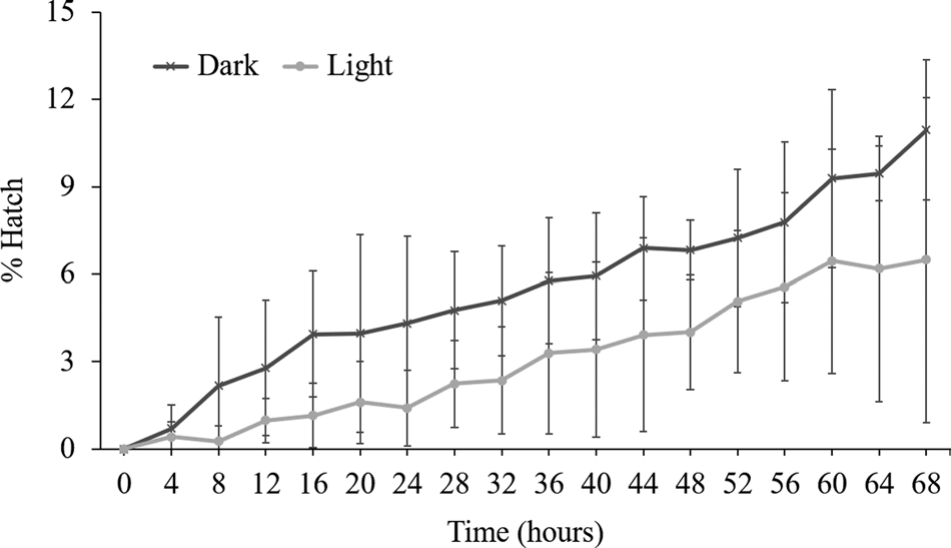
Percent egg hatch is not influenced by light and do not show temporal differences at different time points. Freshly harvested RKN eggs were observed for hatching over a period of 68 hr. Hatched juveniles were counted, and percent hatch was calculated. To examine influence of light we compared percent egg hatch between constant light and constant dark timepoints and to assess any temporal differences, percent egg hatch between consecutive time points under constant dark and constant light were measured. Bars represent standard error and means (*n* =12) are not significantly different (*p* > 0.05) according to paired *t*-test.

To examine if egg hatch exhibits any temporal variation and if it is influenced by light, we numerated egg hatching under constant light and constant dark conditions. There was no difference in the percentage of eggs hatched between consecutive time points (*p* > 0.05) under constant light and constant dark condition (Fig. 6). We also did not observe any statistical differences in the percentage of egg hatch between constant light and constant dark condition revealing no direct effect of light on RKN egg hatch.

### Juvenile mobility is not affected by light

**Figure 7:**
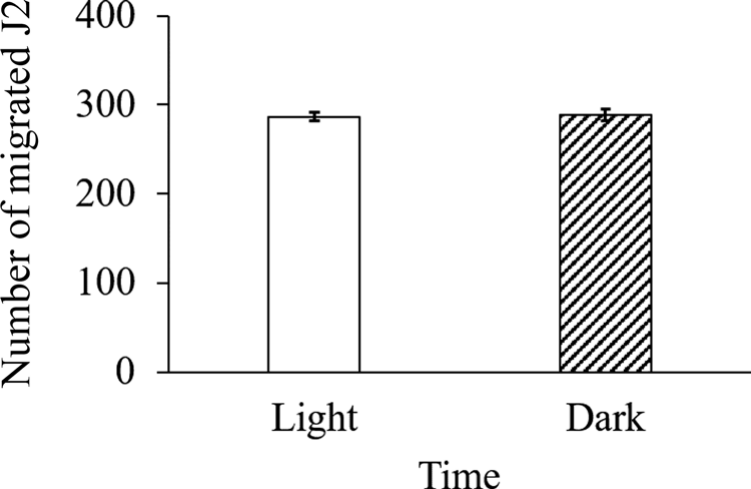
Light does not have influence on RKN juvenile mobility; 300 freshly hatched J2 were placed in a modified Baermann funnel under constant light or constant dark conditions. After 4 hr, the number of J2 that passed through sieve were counted. Bars represent standard error and means (*n* =12) are not significantly different (*p* > 0.05) according to paired *t*-test.

To examine if juvenile mobility is influenced by light, we set up an in vitro trial under constant light and constant dark condition using a mesh sieve and counted nematodes that were able to migrate through the sieve at consecutive time points. There were no significant differences (*p* < 0.05) in the number of J2 passed through the sieve between constant light and constant dark condition (Fig. 7). More than 90% of J2 move through the sieve within 4 hr regardless of light.

## Discussion

Circadian rhythms are based on molecular timekeepers that allow an organism to anticipate daily variation in their environments such as light and temperature. Timekeepers are generally genes that interact with each other and generate oscillations of gene expression. Many plant developmental and physiological processes, including innate immunity, are regulated by this endogenous clock (Yakir et al., 2007; Sharma and Bhatt, 2015). It is possible that obligate parasites, like RKN, are entrained to basic plant biology which shapes their developmental processes. Other plant pathogens, including bacteria and fungi, regulate infection processes and their biology by monitoring host behavior and host cyclic patterns informed by endogenous clocks (Bhardwaj et al., 2011; Wang et al., 2011). Like other plant pathogens, our results also indicate that RKN host seeking and penetration are influenced by plant diurnal and circadian patterning.

In the present study, we demonstrated that RKN penetrate plant roots significantly more when inoculated at night. The greatest susceptibility of the plant to RKN at night suggests that nematodes are more active, more responsive to plant signals, or the host signals are significantly different enough at night than during the day. Through inverted light, egg hatch, and juvenile mobility experiments, our study demonstrated no direct effect of light on nematode biology and revealed a plant host clock-driven phenotype. Light sensing requires the photoreceptors; *Caenorhabditis elegans*, the model free-living soil nematode, have photoreceptors encoded within their genome (Goya et al., 2016). We are unable to locate homologs of *C. elegans* photoreceptor genes encoded in the RKN genomes suggesting that RKN is unable to perceive light.

There are multiple host cues that are implicated in RKN attraction and repulsion. Carbon dioxide has been described as the most potent attractant for RKN (Perry, 2005). The differential expression levels of host cues such as carbon dioxide and some secondary metabolites could be responsible for our observed phenotype of higher RKN penetration at night. Additionally, some flavonoids that have repelling and lethal effects on *M. incognita* J2 (Wuyts et al., 2006; Faizi et al., 2011) follow circadian pattern with lower expression at night (Ni et al., 2018). Other plant–pathogen interactions seem to modulate plant defense pathways according to the circadian clock and this temporal regulation allows the plant to anticipate and respond effectively to pathogens during the day (Bhardwaj et al., 2011). Additionally, low pH has been shown to attract RKN. It is demonstrated that plant roots create an acidic gradient around the roots and acidify the medium to pH 5 or less. The zone of elongation of growing roots is the most acidic region of roots and is most attractive to RKN suggesting that pH gradient are the signals that direct RKN to plant roots (Wang et al., 2009). RKN respond to myriad plant signals including plant volatiles and water-soluble chemical signals to locate host roots (Curtis et al., 2009). There is evidence that plant volatiles are perceived by *M. incognita* and utilized to locate host roots (Kihika et al., 2017). The majority of these phytochemicals are not influenced by diurnal rhythms (Badri et al., 2010). However, there are reports showing an increased flavonoid (Nagasaka et al., 2009; Ni et al., 2018) during the day that has repellent activity toward RKN (Diez and Dusenbery, 1989).

Entraining plants under constant light conditions support our hypothesis that RKN monitor or perceive host biology pre-penetration. Plants, when exposed to constant environmental conditions, are entrained to forced external cues such as temperature and light. This can alter circadian parameters, and in some conditions, can become arrhythmic (Miller, 2004). Our consistent soil and air temperature across time points excludes temperature as a clock entrainment factor. The abolishment of temporal difference in penetration under constant light conditions indicates the RKN monitoring their host. On the other hand, earlier developmental stages of nematode do not follow rhythmic pattern. We found no obvious differences in percentage of egg hatch and juvenile mobility over time points in in vitro condition but around 12% of hatch was observed under both constant light and dark conditions after two days suggesting that *M. incognita* eggs are capable of hatching even without host stimulant, unlike other plant-parasitic nematodes (Wesemael et al., 2006; Thapa et al., 2017).

Identifying that RKN developmental stages not affected by the light shed importance on the host-driven behavior of RKN and demonstrate host biology as a crucial mediator of nematode parasitism. As RKN biology is responsive to host biology, it is possible that important plant pathways involve in host–nematode interaction can be manipulated to develop control measures for this devastating pathogen. This study also illustrated the importance of considering the time of infection while studying these intimate host–pathogen interactions.
